# CTCF promotes epithelial ovarian cancer metastasis by broadly controlling the expression of metastasis-associated genes

**DOI:** 10.18632/oncotarget.19216

**Published:** 2017-07-10

**Authors:** Lintao Zhao, Yang Yang, Shigang Yin, Tao Yang, Jing Luo, Rongkai Xie, Haixia Long, Lubin Jiang, Bo Zhu

**Affiliations:** ^1^ Institute of Cancer, Xinqiao Hospital, Third Military Medical University, Chongqing, China; ^2^ Department of Obstetrics and Gynecology, Xinqiao Hospital, Third Military Medical University, Chongqing, China; ^3^ Key Laboratory of Molecular Virology and Immunology, Institut Pasteur of Shanghai, Chinese Academy of Sciences, Shanghai, China; ^4^ Institute of Cancer, PLA 324 Hospital, Chongqing, China

**Keywords:** CTCF, metastasis, ovarian cancer, metastasis-associated genes, invasion

## Abstract

CCCTC-binding factor (CTCF) functions as both an oncogenic and a tumor suppressor, depending on the cancer type, through epigenetic regulation. Epigenetic regulation plays a key role in cancer metastasis. Our objective was to investigate whether CTCF plays a crucial role in epithelial ovarian cancer metastasis. First, we found that CTCF expression was increased in ovarian cancer tissues compared to non-tumor tissues. Increased expression of CTCF predicts poor prognosis of ovarian cancer patients. In addition, CTCF knockdown significantly inhibited the metastasis of ovarian cancer cells, although it had no effect on cell proliferation and tumor growth. More importantly, CTCF expression was higher in metastatic lesions compared to primary tumors from the same ovarian cancer patients. We also demonstrated that CTCF affects a number of metastasis-associated genes, including *CTBP1*, *SERPINE1* and *SRC*. Additionally, our ChIP-seq results revealed that these genes have multiple CTCF-binding sites, findings that were further confirmed by ChIP-PCR. Our results suggest that CTCF could be a novel drug target to treat ovarian cancer by interfering with cancer cell metastasis.

## INTRODUCTION

Ovarian cancer is one of the most common and lethal gynecological malignancies worldwide [1]. Over 90% of ovarian cancer cases are ovarian epithelial carcinomas [2]. Despite advances in treatment, the five-year survival rate of epithelial ovarian cancer remains at approximately 30% [3]. Moreover, greater than 60% of patients are diagnosed with advanced disease with intraperitoneal or distant metastasis [4]. The cure rate for ovarian cancer patients without or with metastasis is 88% and 18%, respectively [5]. Accordingly, elucidating the mechanisms underlying metastasis in epithelial ovarian cancer will contribute to the development of novel therapeutic strategies and improve prognosis.

Growing evidence indicates that tumor metastasis is mediated through the upregulation of metastasis-promoting genes [6], and the downregulation of metastasis suppressor genes [7]. In addition, besides changes in gene expression, epigenetic changes, including DNA methylation and histone modifications, also play an important role in tumor metastasis. Aberrant DNA methylation is a well-established molecular hallmark of metastatic lesions in various cancers [8, 9]. These methylation changes appear to regulate the expression of multiple genes that promote or suppress metastasis, including *TIMP2* [10], *CDH1* [11], *CXCL12* [12], and *SERPINB5* [13], by regulating events, such as angiogenesis, epithelial-mesenchymal transition (EMT), migration, invasion and extravasation. Histone modifications, especially histone lysine methylation (HKM), are also associated with tumor metastasis. In particular, repressive histone modifications, such as H3K9 and H3K27 methylation, have been reported to inhibit genes, like *SERPINB5* and *DSC3*, which could inhibit metastasis [14]. In ovarian cancer, a series of genes associated with metastasis have been shown to be downregulated by hypermethylation, including *CDH1*, *CDKN2A* and *RARB* [15]. However, the molecular mechanisms of these alterations are poorly understood.

The CCCTC-binding factor (CTCF) is an 11 zinc finger transcription factor that binds more than 20,000 sites in the human genome [16], with the potential to mediate many epigenetic phenomena [17–20]. Previous studies have demonstrated that the expression pattern and role of CTCF vary in different tumor types. Sporadic mutations in CTCF or defective CTCF function have been observed in various cancer types [21–23], with CTCF functioning as a tumor suppressor. Disruption of promoter methylation, chromatin modifications and structural changes mediated by CTCF can lead to the dysregulation of cancer-associated genes, including *RARRES1* [24], *HOXA10* [25], *TERT* [26] and *PTGS2* [27]. In contrast, some studies have suggested that CTCF may function as an oncogene in breast cancer [28] and acute lymphoblastic leukemia (ALL) [29]. However, the expression pattern of CTCF and its roles in ovarian cancer growth and metastasis remain unknown.

Here, we report that CTCF expression was upregulated in ovarian cancer tissues and that increased CTCF expression was directly associated with poor prognosis of epithelial ovarian cancer patients. In addition, to the best of our knowledge, this study is the first to demonstrate that CTCF knockdown significantly suppressed ovarian cancer cell migration and invasion *in vitro* and tumor metastasis *in vivo*. Moreover, CTCF was also found to regulate a variety of metastasis-associated genes, including *CTBP1*, *SERPINE1* and *SRC*. These findings suggest that CTCF may represent a novel therapeutic target for ovarian cancer metastasis intervention.

## RESULTS

### CTCF expression is upregulated in epithelial ovarian cancer and correlates with poor prognosis

**Figure 1:**
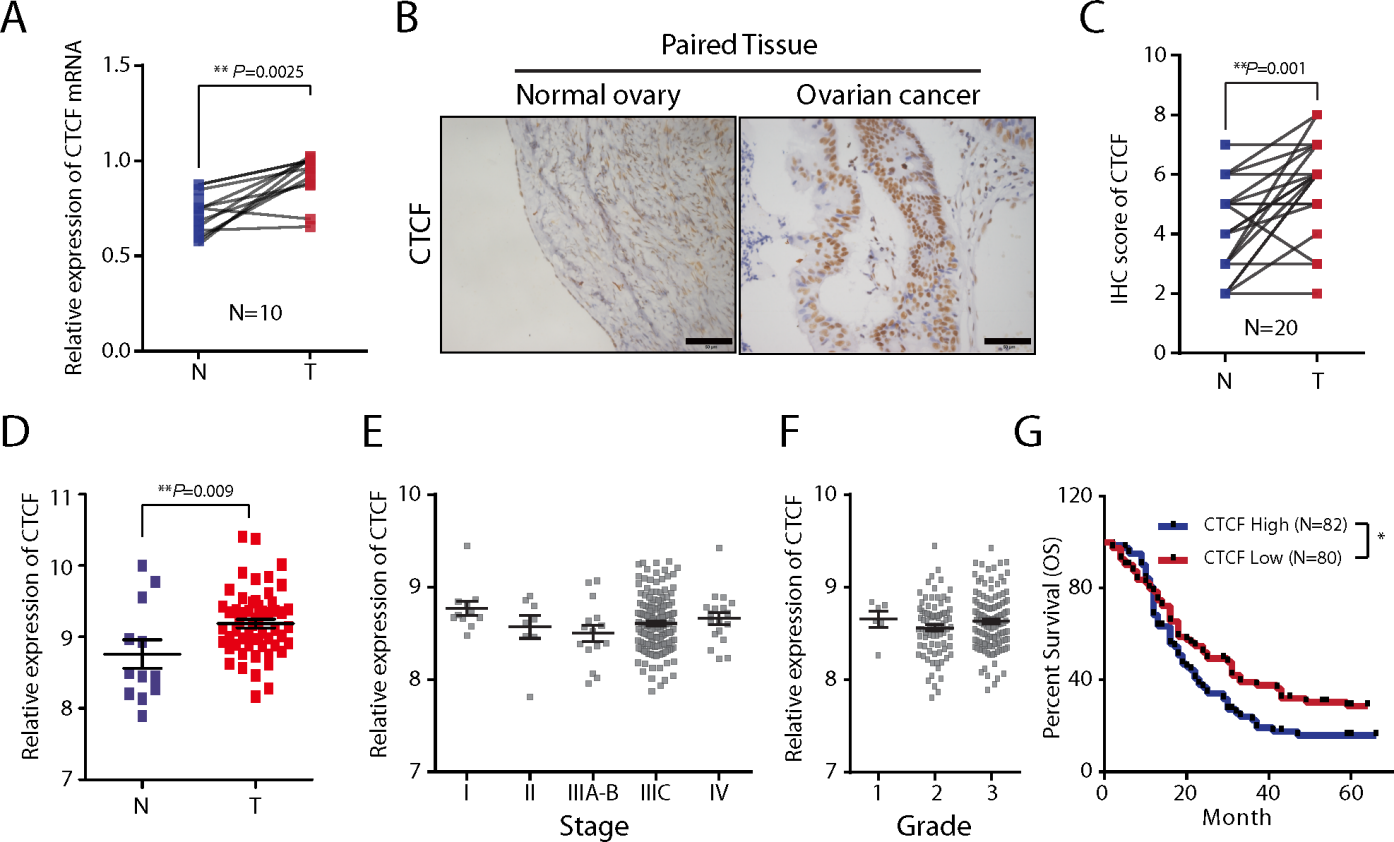
The expression and clinical significance of CTCF in ovarian cancer **(A)** CTCF mRNA expression in ovarian cancer tissues and the paired normal tissues from 10 patients was determined by qPCR analysis. Actin beta (ACTB) was used as a reference gene. N, normal tissue; T, tumor tissue. **(B)** CTCF protein expression was examined by IHC analysis in ovarian cancer tissues and paired normal tissues from 20 patients. Representative images are shown (scale bar, 50 μm). **(C)** The results of the CTCF IHC analysis (B) were scored as described in Materials and methods. The IHC scores were analyzed by paired t-test. N, normal tissue; T, tumor tissue. **(D)** The relative CTCF mRNA expression level was analyzed in 57 ovarian cancer samples and 12 non-tumor samples obtained from the GEO database (GSE66957). **(E-F)** A comparison of the relative expression levels of CTCF in ovarian cancer patients from the GEO database (GSE9891) with different stages (E) and different grades (F) of the disease. **(G)** Kaplan–Meier survival curves of ovarian cancer cases derived from the GEO database (GSE13876, We ranked the survival time of 162 tumor-bearing patients according to the CTCF expression levels, then divided them equally into three groups, and compared the survival time between the highest-expression group and the lowest-expression group). Representative data from 3 independent experiments are shown. ^*^P<0.05, ^**^P<0.01.

We performed qPCR and IHC analyses to examine CTCF expression in epithelial ovarian cancer specimens and paired non-tumor normal tissues from a total of 30 patients. We found that CTCF mRNA expression levels were approximately 2-fold higher in tumor specimens compared with the paired normal tissues (n=10, *P*=0.0025; Figure 1A). The results of the IHC analysis also demonstrated significantly increased expression of CTCF in tumor specimens compared with the matched normal tissues (n=20, *P*=0.001; Figure 1B and 1C). In addition, we observed increased CTCF expression in epithelial ovarian cancer samples (n=57) compared with non-tumor samples (n=12) in a cohort of patients from a GEO database (GSE66957, *P*=0.009; Figure 1D). We additionally found that CTCF mRNA was upregulated in non-small-cell lung carcinoma (NSCLC, *P*=0.0061), gastric cancer (*P*=0.0339) and melanoma (*P*=0.0006), compared with the corresponding non-tumor tissues, by analyzing clinical data obtained from the GSE19804, GSE2685 and GSE8401 GEO databases, respectively (Supplementary Figure 1A-1C).

To investigate the clinical significance of the CTCF expression changes in epithelial ovarian cancer, we analyzed the correlations between *CTCF* expression and tumor stage, tumor grade and 5-year overall survival rate using patient data obtained from the GEO databases GSE9891, GSE9891 and GSE13876, respectively. There were no significant differences in *CTC*F expression associated with different tumor stages (*P*>0.05; Figure 1E) or tumor grades (*P*>0.05; Figure 1F). Noteworthy, we found that *CTCF* expression was inversely correlated with the 5-year overall survival rate of ovarian cancer patients (*P*=0.027; Figure 1G). These data suggest that CTCF may play a role in the process of tumor progression.

### Targeting CTCF in epithelial ovarian cancer cells has no effect on cell proliferation or tumor growth

To further study the function of CTCF in epithelial ovarian cancer progression, we transfected 2 ovarian cancer cell lines (SKOV3 and A2780) with 2 different lentiviral shRNAs. Both shCTCF1 and shCTCF2 decreased CTCF mRNA expression by 60-70% in the 2 cell lines (*P*<0.001; Supplementary Figure 2A). In addition, Western blot analysis and immunofluorescence analysis demonstrated that shCTCF1 and shCTCF2 also significantly reduced the CTCF protein levels (Supplementary Figure 2B-2C).

Unregulated cell proliferation has a well-established link to rapid tumor progression and poor prognosis [33]. Thus, we evaluated the effects of CTCF knockdown on the proliferation of cancer cells. The results of the CCK-8 assay revealed that CTCF silencing did not affect proliferation of SKOV3 or A2780 cells (Supplementary Figure 3A). Likewise, the xCELLigence RTCA system did not detect any changes in cell proliferation after CTCF knockdown at any of the time points evaluated (Supplementary Figure 3B). To further examine whether CTCF silencing affects tumor growth *in vivo*, we subcutaneously injected 1×10^7^ vector- or shCTCF1-transfected SKOV3 cells into nude mice. Consistent with the *in vitro* results, no significant differences were detected in tumor growth between the 2 groups (Supplementary Figure 3C). On day 35 after cell transplantation, no significant differences were observed in tumor volume (Supplementary Figure 3D) and weight (*P*=0.55; Supplementary Figure 3E) between sacrificed mice in the 2 groups. In addition, IHC analysis revealed no differences in the number of PCNA-positive cells in xenografted tumors between the 2 groups (*P*=0.91; Supplementary Figure 3F and Supplementary Figure 3G). Together, these results indicate that CTCF promotes ovarian cancer progression in a cell proliferation-independent manner.

### Targeting CTCF in epithelial ovarian cancer cells suppresses cell migration and tumor metastasis

**Figure 2:**
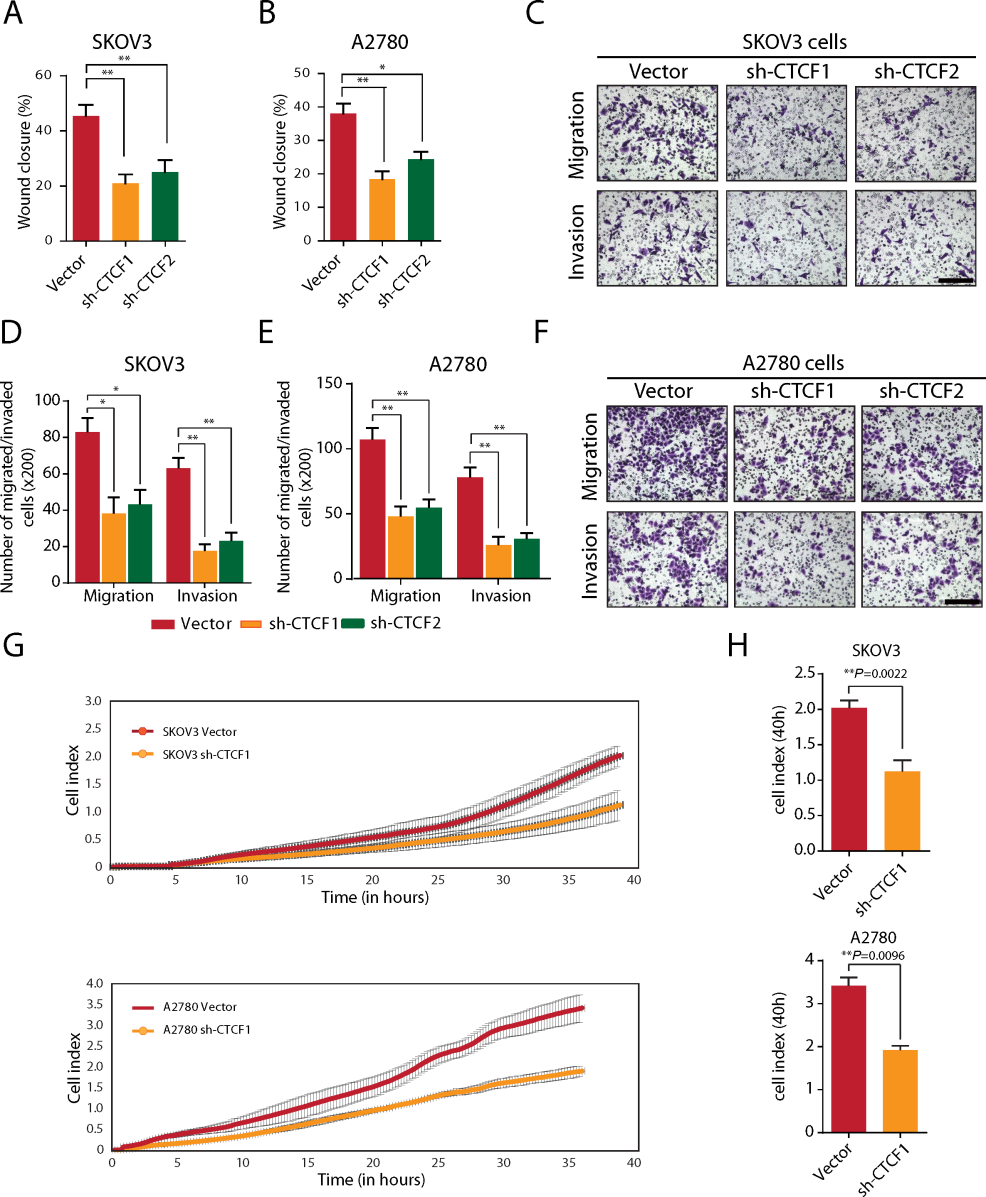
CTCF knockdown inhibits the migration and invasion of ovarian cancer cells *in vitro* **(A-B)** Wound healing assays were performed, and the extent of wound closure in vector- or shCTCF-transfected SKOV3 (A) and A2780 cells (B) was compared. **(C)** The migration and invasion of SKOV3 cells were evaluated using a transwell assay. Representative images are shown (scale bar, 50 μm). **(D)** The result of the statistical analysis of the data obtained from the transwell assay is shown. **(E)** The migration and invasion of A2780 cells was evaluated using the transwell assay. The result of the statistical analysis of the transwell assay data is shown. **(F)** Representative images of cells in (E) are shown (scale bar, 50 μm). **(G)** The RTCA test system was used to evaluate migration of SKOV3 and A2780 cells. Representative curves are shown. **(H)** The cell index in (G) was calculated and analyzed. Representative data from 3 independent experiments are shown. ^*^P<0.05, ^**^P<0.01.

Tumor metastasis is another important determinant of cancer prognosis [34]. Accordingly, we further examined the effects of CTCF knockdown on the migration and invasion of ovarian cancer cells. We found that CTCF silencing significantly impaired the wound healing assay in the SKOV3 cell groups (*P*<0.01; Figure 2A and Supplementary Figure 4A) and the A2780 cell groups evaluated (*P*<0.05; Figure 2B and Supplementary Figure 4B). Furthermore, the transwell assay revealed that CTCF knockdown led to a 50% reduction in the number of migrated cells and a 60-70% reduction in the number of invasive cells (Figure 2C-2F). Consistent with these findings, the xCELLigence RTCA system demonstrated that the index of migrated cells decreased by approximately 50% in shCTCF1-transfected SKOV3 cells (*P*=0.0022) and A2780 cells (*P*=0.0096) compared with the vector-transfected cells at 40 hours (Figure 2G-2H).

**Figure 3:**
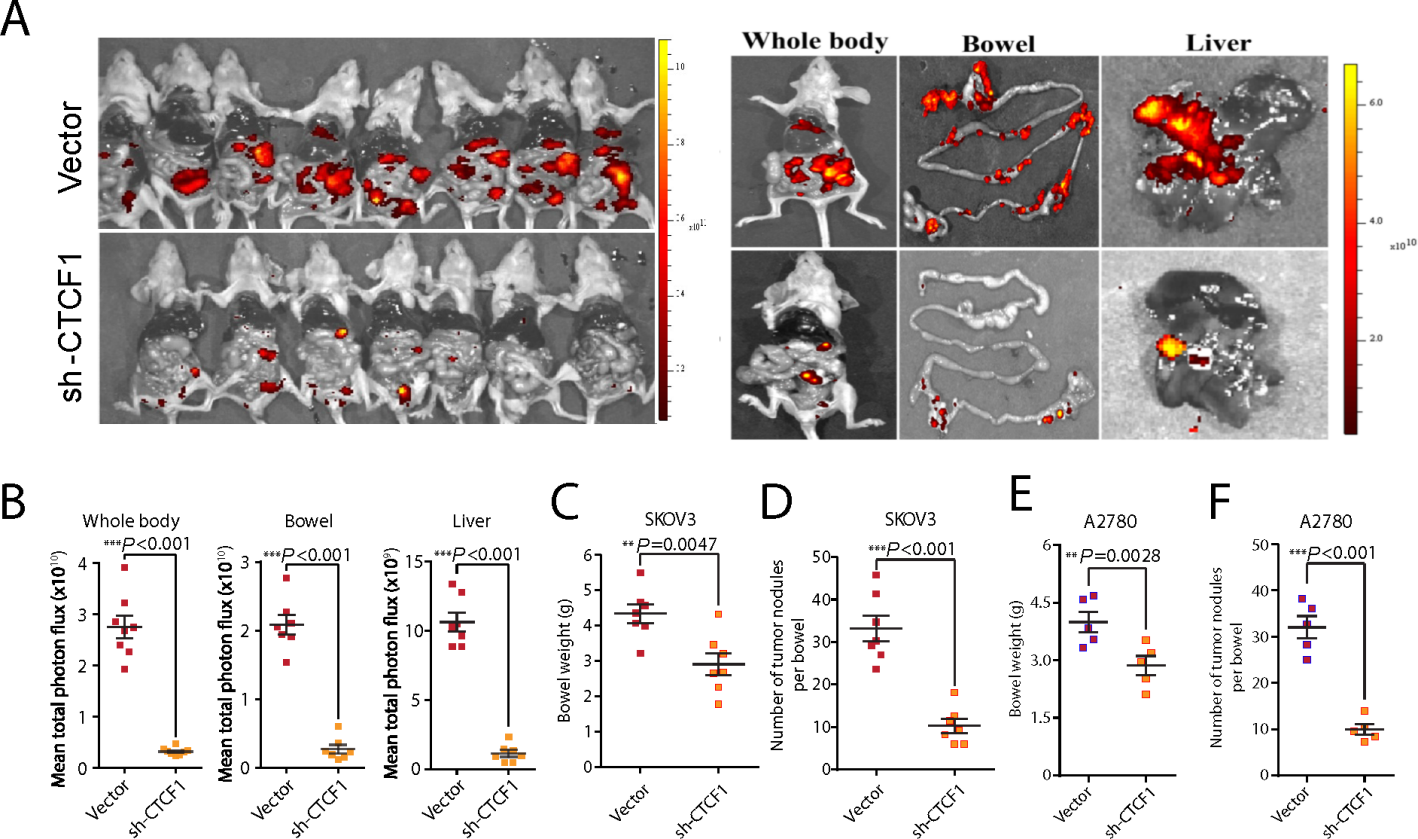
CTCF knockdown suppresses ovarian cancer metastasis *in vivo* A total of 1×10^6^ vector- or sh-CTCF1-transduced SKOV3 cells were injected intraperitoneally into each nude mouse. **(A)** Metastasis was monitored using bioluminescence imaging. Whole-body images of mice on the day they were sacrificed are shown in the left panel. Representative fluorescence images of bowels and livers are shown in the right panel. **(B)** The fluorescence intensity of the whole body, bowel and liver of mice from the 2 groups was statistically analyzed. **(C)** Average bowel weight of the mice in (B) at the time they were sacrificed is shown. **(D)** The average number of tumor nodules per bowel in mice from both groups was calculated and analyzed. **(E-F)** The average bowel weight (E) and number of tumor nodules per bowel (F) in mice injected with vector- or sh-CTCF1- transfected A2780 cells are shown. Representative data from 3 independent experiments are shown. ^**^P<0.01, ^***^P<0.001.

To investigate whether CTCF knockdown impairs ovarian cancer metastasis *in vivo*, a tumor metastasis model was established as previously described [32]. A total of 1×10^6^ shCTCF1- or vector-transfected SKOV3 and A2780 cells were injected intraperitoneally into nude mice. As anticipated, bioluminescence imaging revealed that the CTCF-knockdown SKOV3 cells were associated with significantly fewer metastases compared with vector-transfected cells, especially in the liver and bowel tissues (*P*<0.001; Figure 3A and 3B). Moreover, the bowel weight (*P*=0.0047; Figure 3C) and number of metastatic nodules in the bowels (*P*<0.001; Figure 3D) were significantly decreased in mice transplanted with CTCF-knockdown SKOV3 cells compared with the vector group. Additionally, we observed a substantial attenuation of tumor metastasis throughout the whole body of mice transplanted with shCTCF1-transfected A2780 cells compared to mice transplanted with control cells (Supplementary Figure 4C). Moreover, the bowel weight (*P*=0.0028; Figure 3E) and number of tumor nodules in the bowels (*P*<0.001; Figure 3F) were also significantly reduced in the CTCF-knockdown A2780 group. Taken together, these data suggest that CTCF may play a key role in ovarian cancer metastasis.

### CTCF expression is upregulated in metastatic lesions of human epithelial ovarian cancer

**Figure 4:**
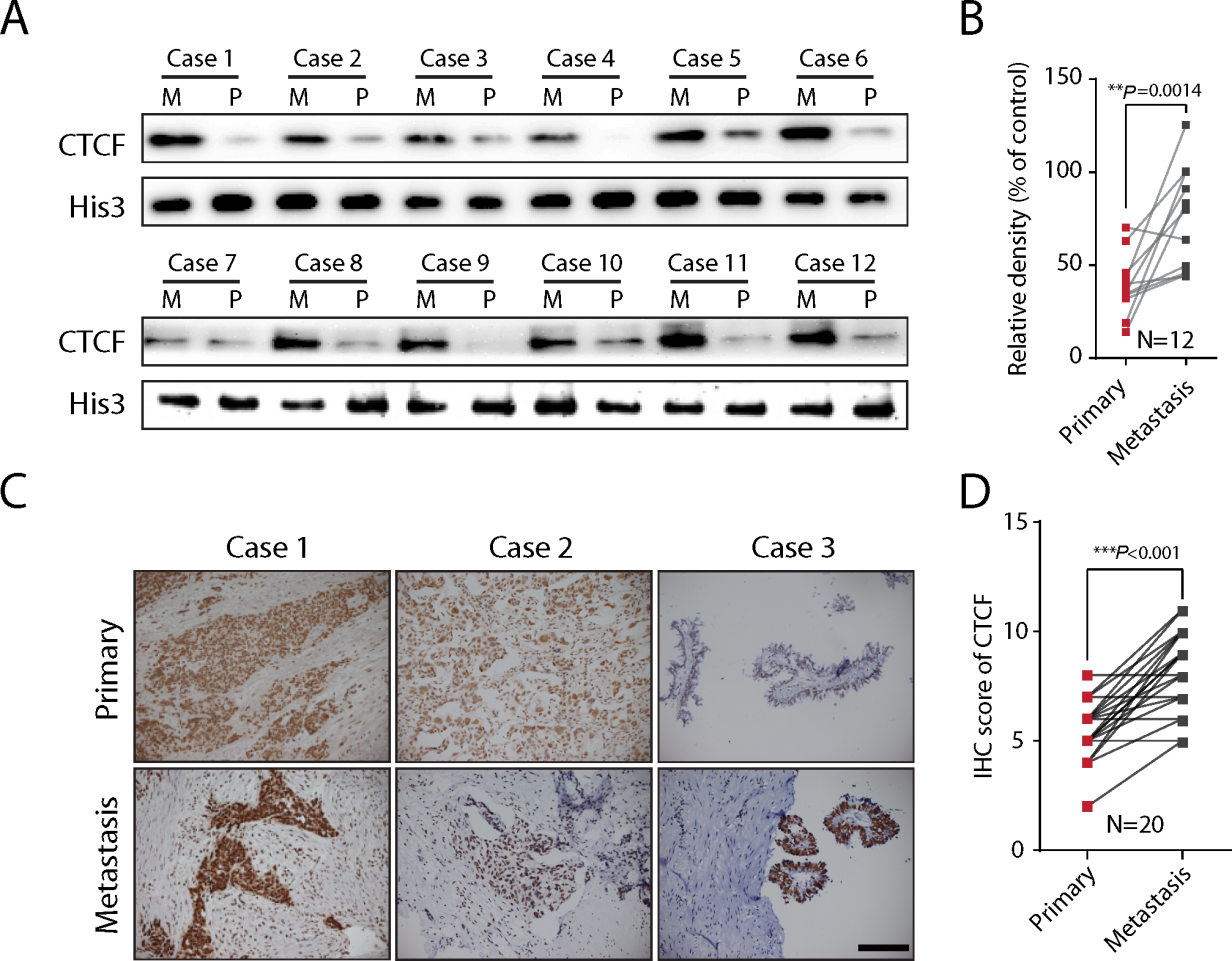
CTCF expression is upregulated in metastatic lesions of ovarian cancer **(A)** CTCF expression was evaluated in metastatic (M) and primary lesions (P) from 12 ovarian cancer patients by Western blot analysis. Histone 3 was used as the reference protein. **(B)** The gray value of CTCF expression in (A) was calculated and analyzed. **(C)** IHC analysis was used to evaluate CTCF expression in metastatic and primary lesions from 20 ovarian cancer patients. Representative images are shown (scale bar, 50 μm). **(D)** CTCF IHC analysis results in (C) were scored and analyzed by paired t-test. Representative data from 3 independent experiments are shown. ^**^P<0.01, ^***^P<0.001.

To further validate the significance of CTCF in ovarian cancer metastasis, we evaluated CTCF expression in primary lesions and paired metastatic lesions from a total of 12 ovarian cancer patients (Supplementary Table 1). Western blot analysis revealed a 2-fold increase in CTCF protein expression in metastatic lesions (M) compared with primary ovarian cancer lesions (P) (N=12, *P*=0.0014; Figure 4A and 4B). Additionally, the expression of CTCF was also evaluated by IHC analysis of primary and metastatic lesions from a different cohort comprising 20 epithelial ovarian cancer patients (Supplementary Table 1). Consistent with the data from the previous cohort, the expression of CTCF, as quantified by the IHC score, was significantly higher in metastatic lesions than in primary lesions (*P*<0.001; Figure 4C and 4D). These *in vivo* clinical data indicated a positive correlation between CTCF expression and ovarian cancer metastasis, further verifying our *in vitro* results.

### CTCF promotes ovarian cancer metastasis by controlling metastasis-associated genes

**Figure 5:**
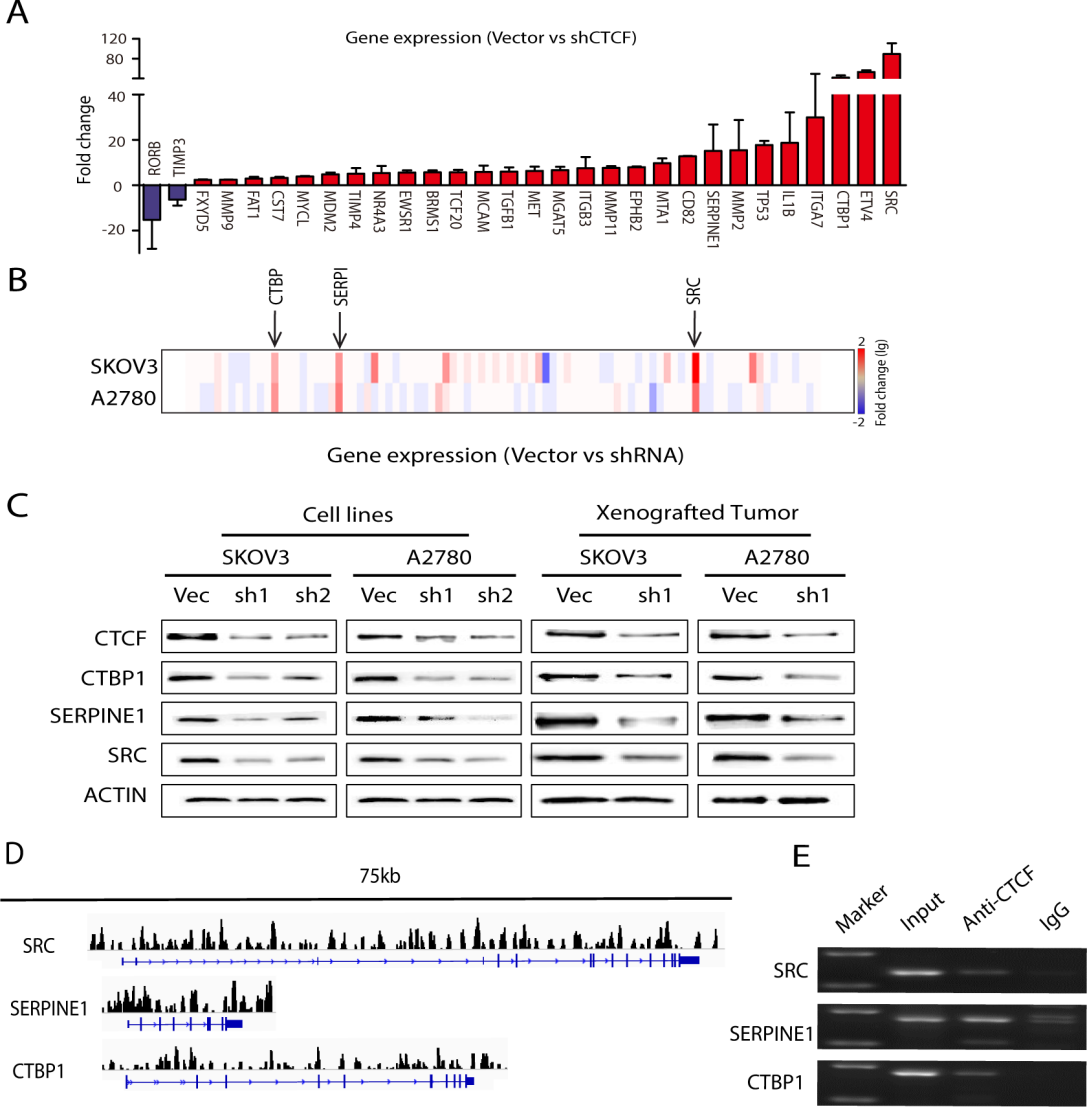
CTCF regulates the majority of the analyzed metastasis-associated genes in ovarian cancer **(A)** PCR-array analysis of 89 genes associated with tumor metastasis was performed to compare the gene expression levels between vector- and shCTCF1-transfected SKOV3 and A2780 cells. Genes whose expression levels changed more than 2-fold are shown. **(B)** The fold-change of 89 genes in **(A)** is expressed as a heat map. **(C)** The expression of CTBP1, SERPINE1 and SRC was examined by Western blot analysis in vector- and shCTCF1-transfected SKOV3 and A2780 cells and in xenografted tumors derived from the metastasis mouse models. Representative data from 3 independent experiments are shown. **(D)** Peak regions of CTCF binding sites for the gene of *SRC*, *CTBP1* and *SERPINE1* are analyzed by Chip-seq in SKOV3 cells. **(E)** CTCF binding sites analyzed by Chip-seq for the gene of *SRC*, *CTBP1* and *SERPINE1* in SKOV3 cells are further confirmed by Chip-PCR, purified input DNA and normal mouse IgG pulldowns are used as control.

To investigate the underlying mechanisms whereby CTCF influences ovarian cancer metastasis, we used PCR-array analysis to compare the gene expression profiles of shCTCF1-transfected and control vector-transfected cancer cells (SKOV3 and A2780). A total of 89 metastasis-associated genes were analyzed, including genes involved in cell adhesion, extracellular matrix (ECM) composition, cell cycle and transcriptional regulation. The PCR-array analysis demonstrated that the expression levels of 30 of the 89 genes evaluated changed more than 2-fold in both shCTCF1-transfected cell lines, of which 28 genes were downregulated and 2 genes were upregulated (Figure 5A and Supplementary Table 2). Most of the downregulated genes, including *SRC* [35], *TGFB1* [36], *MMP9* [32] are known to promote tumor metastasis. In contrast, the 2 upregulated genes, *RORB* [37] and *TIMP3* [38], are suppressors of metastasis. To corroborate the reliability and reproducibility of the screening results, we selected 3 genes (*SRC*, *CTBP1* and *SERPINE*1) for further analysis. The expression of *SRC*, *CTBP1* and *SERPINE1* was decreased more than 10-fold in both groups of CTCF-knockdown cells (Figure 5B). We next verified the expression of the proteins encoded by these 3 genes by Western blot analysis and found that their expression was greatly reduced in both CTCF-knockdown cells lines (Figure 5C, left panel). Moreover, a decrease in the expression of these 3 proteins was also observed in xenografted tumors derived from shCTCF1-transfected cells compared with control vector-transfected cells (Figure 5C, right panel). These data indicate that CTCF may promote ovarian cancer metastasis by broadly regulating the expression of metastasis-associated genes, notably *CTBP1*, *SERPINE1* and *SRC*. In order to further elucidate the details of the underlying mechanisms, we used ChIP-seq technology to identify the genomic locations bound by CTCF in Skov3 cells. We found that 32.7% of the loci were located within intronic regions. 10.3% of the loci were within the promoter region (2 kb upstream of the TSS) (Supplementary Figure 5A-5C). This suggested that binding of CTCF could directly regulate gene expression in ovarian cancer cells. Combined with our PCR-array analysis results, we found that those genes (*SRC*, *CTBP1* and *SERPINE1*) which were increased 10-fold in both the A2780 and SKOV3 control groups compared to the CTCF-shRNA group surprisingly had numerous CTCF binding loci (Figure 5D). Furthermore, to verify the CTCF binding loci that we observed in the ChIP-seq data, we randomly selected a set of binding sites identified by our analysis in those genes (*SRC*, *CTBP1*, *SERPINE1* and *ITGA7*) and performed ChIP-PCR analysis on them using DNA of SKOV3 cells isolated by immunoprecipitation of a sample that was distinct from that used to isolate the DNA used for ChIP-seq. Our ChIP-PCR validations further confirmed the targets identified in those genes (*SRC*, *CTBP1*, *SERPINE1* and *ITGA7*) in the ChIP-seq data (Figure 5E). These results demonstrated that the activated CTCF is capable of directly regulating metastasis-associated gene expression.

### CTCF expression is directly correlated with metastasis-associated genes in human ovarian cancer specimens

**Figure 6:**
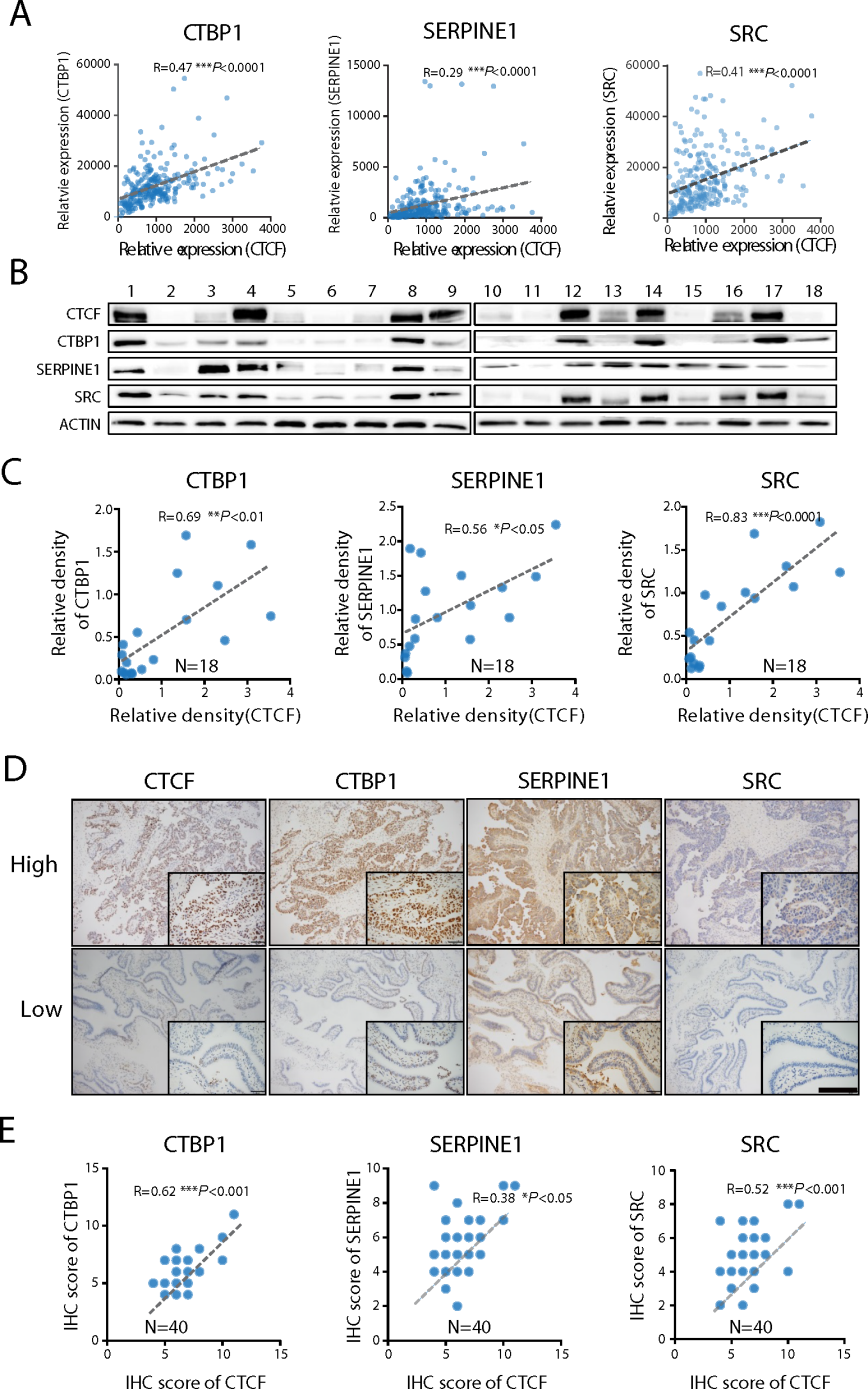
CTCF expression is directly correlated with CTBP1, SERPINE1 and SRC expression **(A)** The correlations between *CTBP1*, *SERPINE1*, *SRC* and *CTCF* in ovarian cancer specimens obtained from the GEO database (GSE13876) were analyzed. **(B)** The expression of CTBP1, SERPINE1, SRC and CTCF in 18 ovarian cancer specimens was determined by Western blot analysis. Representative data from 3 independent experiments are shown. **(C)** The correlations between CTCF expression and CTBP1, SERPINE1 and SRC protein expression in (B) were analyzed. **(D)** The expression of CTBP1, SERPINE1, SRC and CTCF was evaluated by IHC analysis of ovarian cancer specimens from a different cohort comprising 40 patients. Representative images are shown (scale bar, 50 μm). **(E)** The IHC scores were used to analyze the correlations.

To further evaluate the CTCF-mediated regulation of the expression of metastasis-associated genes, we analyzed the correlations between *CTCF* and *SRC*, *CTBP1* and *SERPINE1* gene expression in 255 human ovarian cancer specimens obtained from the GSE13876 database. The results revealed that *CTCF* mRNA expression levels were directly correlated with those of *CTBP1* (R=0.47, *P*<0.001), *SERPINE1* (R=0.29, *P*<0.001) and S*RC* (R=0.41, *P*<0.001) (Figure 6A). Next, we investigated whether a correlation also exists between the CTCF and CTBP1, SERPINE1 and SRC protein levels. Western blot analysis revealed that CTCF protein expression was directly correlated with that of CTBP1 (R=0.69, *P*<0.01), SERPINE1 (R=0.56, *P*<0.05) and SRC (R=0.83, *P*<0.0001) (Figure 6B and 6C) in 18 fresh ovarian cancer specimens (Supplementary Table 3). In addition, we performed IHC analysis to examine the expression of CTCF and SRC, CTBP1 and SERPINE1 in a different cohort comprising 40 ovarian cancer specimens. Consistent with the previous findings, we observed a direct correlation between the expression of CTCF and CTBP1 (R=0.62, *P*<0.001), SERPINE1 (R=0.38, *P*<0.05) and SRC (R=0.52, *P*<0.001) (Figure 6D and 6E). Together, these data strongly suggest that CTCF mediates ovarian cancer metastasis by regulating the expression of metastasis-associated genes, including *CTBP1*, *SERPINE1* and *SRC*.

## DISCUSSION

Currently, the role of CTCF in cancer remains controversial. CTCF is recognized as a putative tumor suppressor, and the disruption of CTCF promotes the development of colorectal cancer [39], prostate cancer [23] and breast cancer [22]. Increased CTCF has been observed in some cancers, including breast cancer [26], ALL [27] and invasive cervical cancer [34], and thus, in these contexts, CTCF possesses oncogenic features. Furthermore, increased levels of CTCF have been associated with resistance to apoptosis in breast cancer [28] and ALL [29]. In the current study, we observed an increase in CTCF mRNA and protein expression in human epithelial ovarian cancer tissues compared with paired normal tissues in 3 cohorts of patients. In addition, we observed increased CTCF expression in NSCLC, gastric cancer and melanoma tissues compared with the matching normal tissues. Accordingly, the nature of CTCF aberrations varies in different types of cancer. More importantly, we found that CTCF expression was an indicator of poor prognosis in ovarian cancer patients. Indeed, our data suggest that CTCF may be an oncogene in epithelial ovarian cancer. However, the significance of CTCF expression in NSCLC, gastric cancer and melanoma remains to be elucidated.

CTCF is a transcription factor reported to be involved in various cellular processes and to play a particularly significant role in cell proliferation. In this study, we did not observe any effects of CTCF knockdown on the proliferation of ovarian cancer cells *in vitro* or *in vivo*. These results are inconsistent with most previous studies. Some studies have reported that CTCF can slow cell cycle progression, disrupt cell division and inhibit cell growth by regulating various growth-related genes in multiple types of cancers [40]. Other studies have reported that CTCF suppresses cell proliferation by inhibiting the NF-κB pathway in ALL [29]. On the other hand, in a recent study, neither ectopic expression nor silencing of CTCF affected the proliferation of liver cancer cells and nasopharyngeal carcinoma cells [41], results that are consistent with the findings of the present study. Together, these data indicate that CTCF exerts different effects on cell proliferation in a cell-type specific manner. However, the underlying mechanism whereby CTCF functions in different contexts requires further investigation.

In addition to tumor growth, tumor metastasis is also an established prognostic factor in cancer [34]. Thus, we hypothesized that CTCF may regulate ovarian cancer metastasis and serve as a prognostic indicator. Consistent with this hypothesis, we found that CTCF promoted cell migration and invasion in ovarian cancer *in vitro* and *in vivo* by loss-of-function studies. We also verified the significance of CTCF in ovarian cancer metastasis in clinical specimens and found that CTCF increased cell invasion and promoted tumor metastasis. A recent study reported that CTCF promoted cell invasion and tumor metastasis in neuroblastoma [42], which further supports our findings. However, it has also been reported that in a CTCF haploinsufficient mouse model, CTCF knockdown promoted invasion, metastasis and EMT in lung, liver and skin cancer [43]. These findings indicate that the role of CTCF in tumor metastasis may also be cell type-specific, genetic background and other variables.

Previous studies have reported that a variety of genes regulated by CTCF, including *RB1* [44], *TERT* [45] and *BAX* [28], are involved in proliferation and apoptosis in cancer cells. However, the mechanism whereby CTCF regulates metastasis-associated genes remains largely unknown. Thus, we conducted a comprehensive investigation to identify metastasis-associated genes regulated by CTCF using PCR-array analysis. We found that the expression of a series of metastasis-associated genes was effected by CTCF knockdown. In particular, the results of our PCR-array suggested that CTCF suppresses the expression of *RORB* and *TIMP3*, which are genes that inhibit metastasis in prostate cancer [37] and breast ductal cancer [38]. Furthermore, genes reported to promote tumor metastasis in various cancers, including *SRC* [35], *TGFB1* [36] and *MMP9* [32], were upregulated by CTCF. In this study, three genes, namely *CTBP1*, *SERPINE1*, and *SRC*, whose expression levels were altered by CTCF exhibiting a change greater than 10-fold were selected for further analysis. *SRC* [35] and *SERPINE1* [46] are reported to be essential for invasion and metastasis in ovarian cancer, phenomena that are associated with a poor prognosis [47]. *CTBP1* mediates metastasis in multiple cancers, including prostate cancer [48], breast cancer [49] and melanoma [50], but its role in ovarian cancer metastasis has not been reported. Indeed, we observed a direct correlation between *CTCF* and *CTBP1*, *SERPINE1*, and *SRC* expression in cell lines, xenografted tumors and human ovarian cancer specimens. Collectively, our data indicate that CTCF may promote ovarian cancer metastasis by broadly regulating a series of metastasis-associated genes.

CTCF genomic interactions remain largely unclear between different cell types or species. To the best of our knowledge, they have not been studied in ovarian cancer cells. Accordingly, in order to further elucidate the details of the underlying mechanisms, we used ChIP-seq technology to identify the genomic locations bound by CTCF in Skov3 ovarian cancer cells. Previous research showed that a large number of CTCF binding sites are intragenic and CTCF is important for maintaining proper expression of target genes, as CTCF depletion reduced expression level of the target genes [51]. These findings are highly consistent with the results of our studies. Our results show that most CTCF binding loci in the Skov3 cell line are located in the intragenic region. We found that 32.7% of the loci were located within introns, and 10.3% of the loci were located within the promoter region (2 kb upstream of the TSS). Together with our results of the PCR-array analysis, we discovered that the genes (*SRC*, *CTBP1* and *SERPINE1*) which were sharply decreased in both A2780 and SKOV3 cells after knockdown of CTCF expression had numerous CTCF binding loci. These suggest that binding of CTCF in ovarian cancer cells could directly regulate a series of metastasis-associated genes.

In summary, we demonstrated that CTCF is overexpressed in ovarian cancer and is associated with a poor prognosis in ovarian cancer patients. CTCF knockdown significantly inhibited cell migration, invasion and tumor metastasis. Our data further indicate that CTCF-mediated metastasis may result from the regulation of a series of metastasis-associated genes, including *CTBP1*, *SERPINE1* and *SRC*. These findings suggest that CTCF may serve as a novel therapeutic target for ovarian cancer treatment.

## MATERIALS AND METHODS

### Patient samples

Fresh surgical specimens were collected from patients with ovarian cancer during surgery at Xinqiao Hospital, and included primary tumor, paired metastatic tissue, and paired non-tumor tissues. The patient details are provided in Supplementary Table 1 and Supplementary Table 2. The paraffin-embedded human specimens were obtained from the Department of Pathology of Xinqiao Hospital. This study was approved by the institutional review board of Xinqiao Hospital, and informed consent was obtained from all individuals.

### Cell culture

Human epithelial ovarian cancer cell lines SKOV3 and A2780 were obtained from the American Type Culture Collection (ATCC, Manassas, VA, USA), and the authenticity of their identity was recently tested. Cells were cultured in DMEM/H (Hyclone, GE Healthcare Bio-Sciences, Pittsburgh, PA, USA) containing 10% fetal bovine serum (Gibco, Grand Island, NY, USA) and 1% penicillin/streptomycin (Beyotime Biotechnology) and incubated at 37°C in a humidified atmosphere with 5% CO_2_.

### Lentivirus transfection

The CTCF-shRNA lentivirus was obtained commercially (Genechem, Shanghai, China). Briefly, a stem-loop oligonucleotide structure containing the CTCF-target sequences was cloned under the control of the human U6 promoter in lentiviral vectors with a GFP reporter. The following sequences were used: shCTCF1, 5’-GTGTCTAAAGAGGGCCTTG-3’ and shCTCF2, 5’-CTGCCACAGATGCCCCCAAC-3’. The lentivirus was transfected into cells following the manufacturer’s protocol. Briefly, cells were dissociated into single cells prior to the day of transduction, and shRNA was added to the medium. After 48 hours, the medium was replaced, and cells were harvested for additional experiments. The efficiency of the CTCF-shRNA knockdown was evaluated using immunofluorescence, qRT-PCR and Western blot analysis.

### Cell counting kit (CCK)-8 assay

A total of 2×10^3^ cells were seeded in 96-well plates and incubated for either 24, 48, 72, 96 or 120 hours. Then, 10 μl of CCK-8 reagents were added to each well and incubated at 37°C for 1 hour, and the optical density (OD) values at 450 nm were measured with Varioskan Flash system (Thermo Fisher, Waltham, MA, USA).

### Wound healing assay

Viable cells (2×10^4^) were plated in 6-well dishes and allowed to form confluent monolayers. The monolayers were incubated in serum-free-medium for 16 hours, and a linear wound was introduced using a 200-μl sterile tip. Wounded monolayers were washed with phosphate buffered saline (PBS), and complete medium was added. The wound was photographed using computer-assisted morphometry (MCID, Cambridge, UK) every 6 hours, and the area was analyzed using the Image J software (http://rsb.info.nih.gov/ij/). The percentage of wound closure was calculated using the following formula: %wound closure = final area/initial area ×100%.

### Transwell assay

A total of 4×10^4^ cells in 200 μl serum-free medium were added to the upper chamber of 24-well culture inserts with a porous polycarbonate membrane that was (8.0 μm, Millipore, Billerica, MA, USA) pre-coated (for invasion) with 30 μl Matrigel (BD Biosciences, San Jose, CA, USA) or not pre-coated (for migration) for 3 hours, and complete media was placed in the lower chamber. The plates were incubated for 24 hours at 37°C in 5% CO_2_. After removing the cells from the upper surface of the membrane with a cotton swab, cells on the lower surface were stained with crystal violet sulfate and counted under a microscope (Olympus, Tokyo, Japan).

### xCELLigence^®^ real-time cell analysis (RTCA) test system

For the cell proliferation assay, 2×10^3^ cells in 100 μl of medium were seeded in each well of the E-plates, and cell proliferation was monitored every 15 minutes for 120 hours using the xCELLigence system (ACEA Biosciences, San Diego, CA, USA). For the cell migration assay, the upper chamber of the CIM-plates was first coated with 1μg/μl of fibronectin. A total of 4×10^4^ cells in 100 μl of serum-free media were seeded in each well of the upper chamber, and 100 μl of complete media was added to each well of the lower chamber. The CIM-plates were left in an incubator for 1 hour to allow for cell attachment and were automatically monitored by the xCELLigence system for 48 hours. The results were expressed as cell index (CI) values. The normalized CI was calculated by dividing the CI at the normalized time by the original CI.

### Immunohistochemistry (IHC) analysis

Paraffin-embedded tissue sections were de-paraffinized with xylene, graded ethanol and ddH_2_O. Antigen retrieval was achieved by incubating the specimens with boiled citrate buffer (10 mM, pH 6.0) for 15 minutes. The tissue sections were subsequently exposed to 3% hydrogen peroxide for 10 minutes to inhibit endogenous peroxidase activity. After blocking with goat serum for 20 minutes at room temperature, the sections were incubated at 4°C overnight with the following primary antibodies: anti-CTCF (Abcam, Cambridge, UK), anti-Ki-67 (Bioss Antibodies Inc., Woburn, MA, USA), anti-SRC (Abcam), anti-CTBP1 (Abcam), and SERPINE1 (Abcam). Then, the sections were incubated with HRP-labeled secondary antibodies at 37°C for 20 minutes and visualized with the DAB detection kit following the manufacturer’s instructions. The negative control samples were incubated with PBS in the absence of primary antibodies. The images were recorded with MCID, and the expression of positive cells was scored as previously described [30]. Briefly, each sample was assigned an intensity score (0-3) and a percentage of positive cells score (0=0%, 1=1-19%, 2=20-79%, 3=80-100%). An overall IHC score was calculated by multiplying the intensity score and the percentage value of positive cells.

### Immunofluorescence

Cells were attached to slides, and after fixation with a 4% paraformaldehyde solution for 30 minutes, the slides were incubated with TritonX-100 for 30 minutes. Then, the slides were blocked with 5% BSA and incubated with anti-CTCF (Abcam) overnight at 4°C. After incubating with Cy3-conjugated IgG (Beyotime Biotechnology, Shanghai, China) for 30 minutes at 37°C, nuclei were counterstained with 4,6-diamidino-2-phenylindole (DAPI). Stained cells were visualized using an Olympus confocal microscope (Olympus) equipped with a Leica camera (Leica).

### Quantitative real-time PCR

Total RNA from cells or tissues was extracted as previously described [31]. After reverse transcription with a cDNA Reverse Transcription Kit (Takara, Tokyo, Japan), quantitative real-time PCR was performed using SYBR Green PCR Mix (Applied Biosystems, Foster City, CA, USA) and IQ5 detection system (Bio-Rad, Hercules, CA, USA). The relative gene expression was calculated with the delta-CT value, and GAPDH was used as the reference gene. The sequences of the primers were as follows: forward, 5’-GTCACCCTCCTGAGGAATCA-3’; reverse, 5’-CGTAATCGCACATGGAACAC-3’.

### Western blot analysis

Total protein was extracted using the RIPA buffer with 1% PMSF (Beyotime Biotechnology). For the Western blot analysis, PVDF membranes were incubated with the following primary antibodies overnight at 4°C: anti-CTCF (Cell signaling Technology, Danvers, MA, USA), anti-Histone3 (Beyotime Biotechnology), anti-SRC (Abcam), anti-CTBP1 (Abcam), anti-SERPINE1 (Abcam) and anti-β-actin (Beyotime Biotechnology). After the samples were washed with 0.1% TBST, they were incubated with the appropriate secondary antibodies (Beyotime Biotechnology), and the Chemilmanger™ 5500 system (Alpha Innotech, San Jose, CA, USA) was used to visualize the results. The gray value was calculated using the Image J software (http://rsb.info.nih.gov/ij/).

### *In vivo* xenograft experiments

Female nude mice were purchased from the Chinese Academy of Medical Sciences (Beijing, China). Mice were housed in laminar flow cabinets under specific pathogen-free conditions. For the experiments evaluating tumor growth, 1×10^7^ cells were resuspended in 150 μl PBS and subcutaneously injected into the right flanks of the mice. The tumor volumes were measured every day. The tumor metastasis model was established as previously described [32]. Briefly, 1Χ10^6^ cells resuspended in 150 μl of PBS were intraperitoneally injected into nude mice. Metastasis was monitored using bioluminescence imaging IVIS200 system (Xenogen Biosciences, Cranbury, NJ, USA). The tumor, liver, spleen, kidney, and bowels of xenografted mice were harvested for further evaluation. The experimental procedures described in this study were approved by the Institutional Animal Care and Use Committee.

### PCR-array

The expression of 89 metastasis-associated genes was analyzed using the Human Tumor Metastasis PCR Array (PAHS-028Z; SABiosciences, QIAGEN, Frederick, MD, USA; http://www.sabiosciences.com/) following the manufacturer’s protocol.

### Chromatin immunoprecipitation and massive parallel sequencing

Chromatin immunoprecipitation (ChIP) was performed as previously described. Briefly, SKOV3 cells were rinsed with room temperature PBS before being crosslinked in a 1%formaldehyde solution. SKOV3 Cells were then harvested and homogenized in the presence of protease inhibitors before DNA was sonicated. Magnetic Dynal beads (Invitrogen, San Diego, CA, USA) combined with a mixture of antibodies (anti-CTCF, Cell Signaling Technology) was used to pull down CTCF overnight. Preimmune serum or IgG were used as negative controls. Sequencing libraries were generated for massive parallel sequencing using standard methods. Briefly, 500 ng of pulldown DNA was subjected to end repair, terminal adenylation, and adapter ligation before fragments ranging from, 400–500bp were isolated from a 2% E-gel (Invitrogen). After a standardized 12 cycle PCR, DNA quality was evaluated on a DNA 1000 Bioanalyzer chip (Agilent Technologies, Santa Clara, CA, USA) before being submitted for sequencing on an Illumina GAII (Illumina, Inc., San Diego, CA, USA). All ChIP-seq data is deposited in the Gene Expression Omnibus (GEO) database at the National Center for Biotechnology Information (http://www.ncbi.nlm.nih.gov/geo/query/acc.cgi?acc=GSE8545) and their accession number is: GSE85453.

### ChIP-PCR

Our CTCF ChIP-seq results were confirmed via ChIP-PCR. Briefly primers of approximately 150 bp were constructed to cover regions that were sequenced in the ChIP-seq experiment, and amplified with SYBR Green (Millipore, Upstate Biotechnology, Inc. Lake Placid, NY, USA). DNA of SKOV3 cells was quantified on a nanodrop ND3300 (Thermo Scientific, Waltham, MA) using a Quant-iT Picogreen dsDNA Assay Kit (Invitrogen), and equal quantities of DNA were used as template. Comparison of input, CTCF and IgG pulldowns are reported as the average according to the manufacturer’s protocol (Millipore, Upstate Biotechnology, Inc.). For a complete list of the primers used to amplify those regions see Supplementary Table 4.

### Statistical analysis

All quantitative data were expressed as the mean ± SEM. The independent samples t-test, Cox regression analysis and Pearson correlation analysis were used to conduct the statistical analyses. Differences were considered statistically significant when *P*<0.05. Statistically significant *P* values are presented as *P<0.05, **P<0.01, and ***P<0.001. All statistical analyses were carried out using SPSS 18.0 software (IBM Corp., Armonk, NY, USA; http://www.spss.com.cn/). All experiments were repeated at least 3 times.

## SUPPLEMENTARY MATERIALS FIGURES AND TABLES




