# Supplementary material for: CTCF promotes epithelial ovarian cancer metastasis by broadly controlling the expression of metastasis-associated genes
Source: Oncotarget. 2017 Jul 10;8(37):62217–30. doi: 10.18632/oncotarget.19216 (PMC5617499; doi:10.18632/oncotarget.19216)
Supplement: Supplementary file 1 [file oncotarget-08-62217-s001.pdf]

CTCF promotes epithelial ovarian cancer metastasis by broadly controlling the expression of metastasis-associated genes

SUPPLEMENTARY MATERIALS

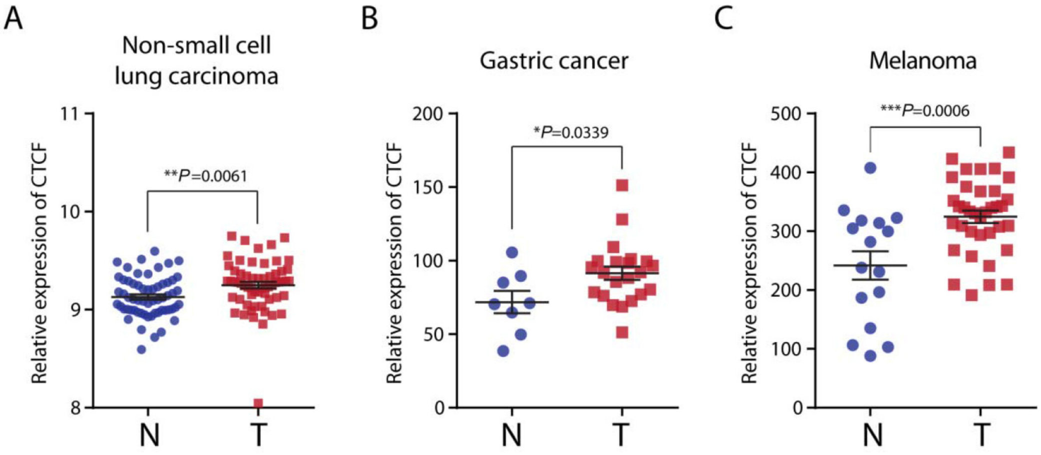

Supplementary Figure 1: CTCF expression in other malignancies. **(A-C)** CTCF mRNA expression was analyzed in Non-small cell lung carcinoma **(A)**, gastric cancer **(B)** and melanoma **(C)** from GEO database (GSE19804, GSE2685 and GSE8401).

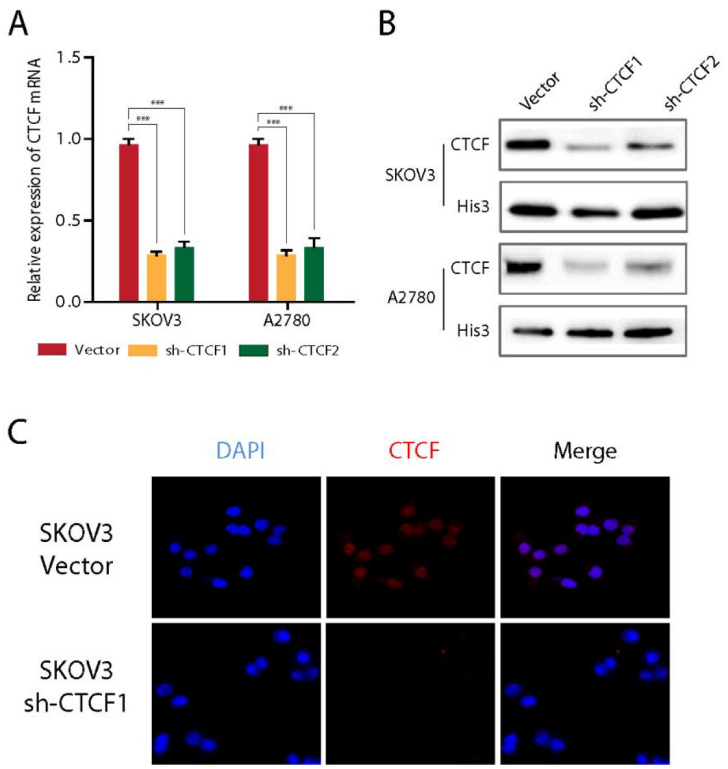

Supplementary Figure 2: CTCF knockdown in ovarian cancer cells. (A) CTCF mRNA expression in vector- or shCTCF-transfected SKOV3 and A2780 cells was evaluated using qPCR. **(B)** CTCF protein expression was assayed in vector- or shCTCF-transfected SKOV3 and A2780 cells. **(C)** Immunofluorescence staining of CTCF in vector- or shCTCF1-transfected SKOV3 cells.

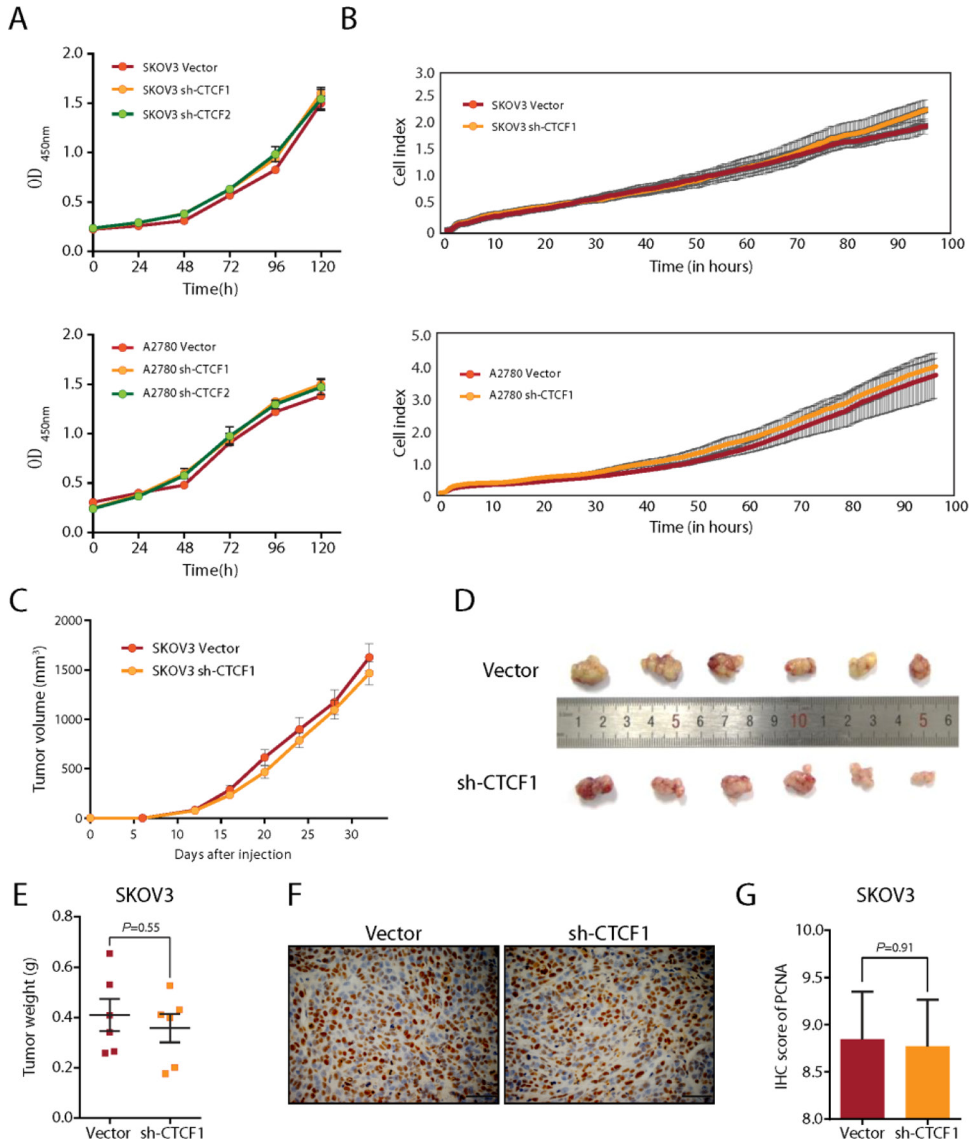

Supplementary Figure 3: The effects of CTCF knockdown on ovarian cancer cell proliferation and tumor growth. (A) Cell proliferation was evaluated using the CCK-8 assay in vector- or shCTCF-transfected SKOV3 and A2780 cells. **(B)** Cell proliferation was evaluated in vector- or shCTCF-transfected SKOV3 and A2780 cells using the xCELLigence RTCA system. **(C)** A total of 1×10^7 vector- or shCTCF1-transfected SKOV3 cells were subcutaneously injected into each nude mouse, and the tumor growth was monitored. **(D)** Representative images of xenografted tumors on the day the mice were sacrificed. **(E)** Weights of xenografted tumors on the day the mice were sacrificed. **(F-G)** IHC staining for PCNA in xenografted tumors in the 2 groups.

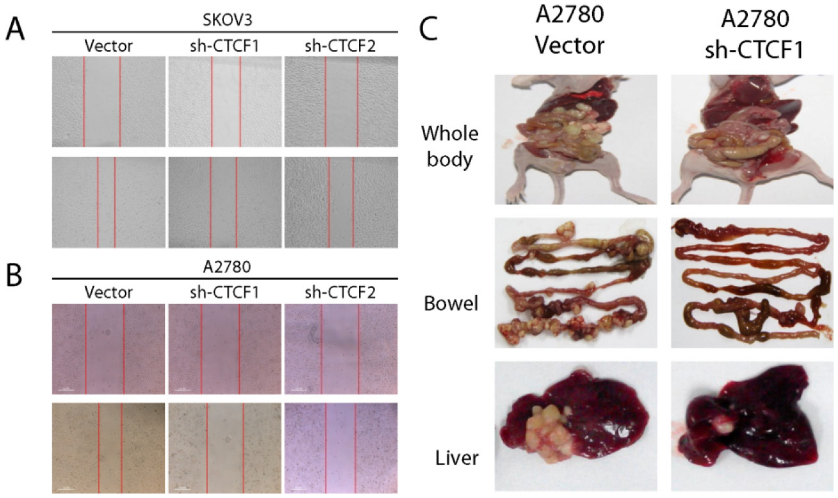

Supplementary Figure 4: The effects of CTCF knockdown on ovarian cancer cell migration and tumor metastasis. (A-B) Representative images of the wound healing assay results at 48 hours in vector- or sh-CTCF1 transfected SKOV3 **(A)** and A2780 cells **(B)** are shown. **(C)** 1×10^6 vector- or shCTCF1-transfected A2780 cells were intraperitoneally injected into each nude mouse. Representative images of the abdominal cavity, bowel and liver of nude mice on the day they were sacrificed are shown.

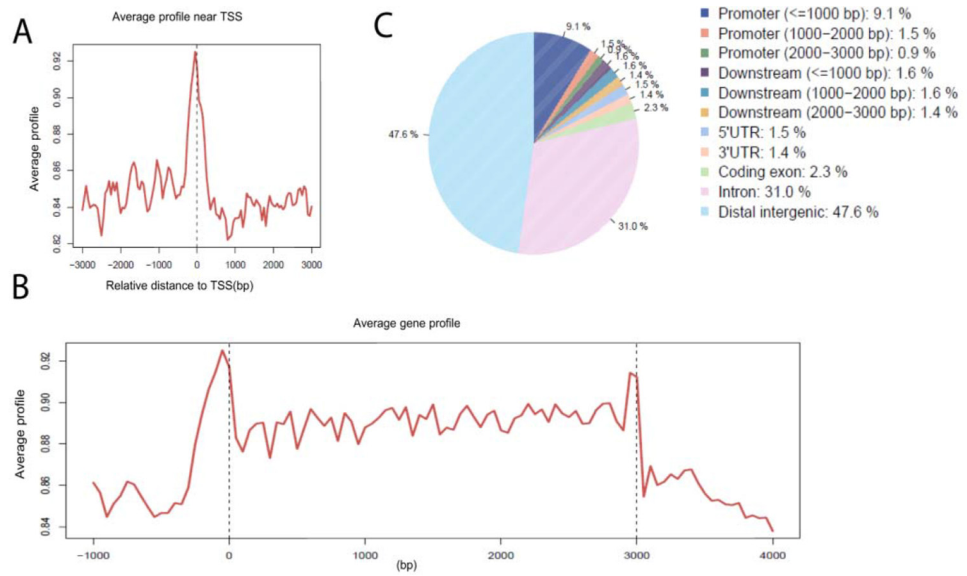

Supplementary Figure 5: Identification of CTCF binding loci. **(A)** The position of CTCF-binding sites with respect to the nearest TSS was plotted as a frequency distribution using 3-kb windows. **(B)** The distribution of the location of CTCF binding loci based on their relative to a closest known RefGene 5'TSS. **(C)** Genomic location of CTCF binding sites identified by ChIP-Seq.

Supplementary Table 1: The information associated with the 20 patients with metastatic ovarian cancer from the study comparing CTCF expression between primary loci and metastatic loci (Figure 4)

No.	Histology	Primary ovary loci	Metastatic loci	Analysis
1	Serous	left	lymph nodes	WB, IHC
2	Serous	bilateral	omentum	WB, IHC
3	Serous	bilateral	omentum	WB, IHC
4	Serous	bilateral	omentum	WB, IHC
5	Serous	left	lymph nodes	WB, IHC
6	clear cell	bilateral	omentum	WB, IHC
7	Serous	right	omentum	WB, IHC
8	Serous	bilateral	omentum	WB, IHC
9	Serous	right	omentum	WB, IHC
10	Endometrioid	bilateral	omentum	WB, IHC
11	Serous	left	omentum	WB, IHC
12	Serous	bilateral	omentum	WB, IHC
13	Serous	left	omentum	IHC
14	Serous	left	omentum	IHC
15	Serous	right	omentum	IHC
16	Endometrioid	bilateral	omentum	IHC
17	Serous	left	omentum	IHC
18	Serous	bilateral	omentum	IHC
19	Serous	right	omentum	IHC
20	Serous	bilateral	omentum	IHC

Supplementary Table 2: Differences in gene expression profiles related to tumor metastasis between the vector- and shCTCF-tansfected SKOV3 and A2780 cells

See Supplementary File 1

Supplementary Table 3: The information associated with the18 ovarian cancer patients from the study examining CTCF, SRC, CTBP1 and SERPINE1 expression (Figure 6B)

No	Age	Histology	Stage	Differentiaiton
1	58	Serous	II	low
2	46	Serous	II	high
3	48	Serous	III	low
4	66	Serous	II	high
5	53	Serous	IIIb	low
6	43	Serous	IIIa	low
7	53	Serous	IIIc	low
8	46	Serous	IIIc	high
9	41	Serous	II	low
10	39	Serous	IIIb	low
11	58	Serous	II	low
12	64	Serous	IIIa	medium
13	58	Serous	IIIc	low
14	55	Serous	IIIb	low
15	67	Serous	IIIc	low
16	49	Serous	II	medium
17	52	Serous	IIIa	low
18	68	Serous	IIIa	high

Supplementary Table 4: A list of primers designed for ChIP-PCR

Gene	Forward	Reverse
SRC	**GCCATAAAGCGAGGTTCATA**	**AAGTGTGCCCTAAGTGTTTG**
SERPINE1	**AAGGCTATTGGGGTTTGCT**	**GATGTGGGCAGGAAATAGATG**
CTBP1	**ACACCCAAGGTCCTCAG**	**TCCTTTGTTCACAGAGTCAG**
