# Supplementary material for: CTCF promotes epithelial ovarian cancer metastasis by broadly controlling the expression of metastasis-associated genes
Source: Oncotarget. 2017 Jul 10;8(37):62217–30. doi: 10.18632/oncotarget.19216 (PMC5617499; doi:10.18632/oncotarget.19216)
Supplement: Supplementary file 2 [file oncotarget-08-62217-s002.docx]

**Supplementary Table 2: Differences in gene expression profiles related to tumor metastasis between the vector- and shCTCF-tansfected SKOV3 and A2780 cells**

| **Symbol** | **Fold change** | **Fold change** |
| --- | --- | --- |
|  | **A2780** | **SKOV3** |
| APC | 2.56 | 1.06 |
| BRMS1 | 6.56 | 4.78 |
| CCL7 | -3.01 | 1.59 |
| CD44 | -1.48 | 4.64 |
| CD82 | 12.90 | 12.58 |
| CDH1 | 7.02 | 1.78 |
| CDH11 | -1.48 | -1.38 |
| CDH6 | 3.72 | -1.08 |
| CDKN2A | -1.07 | -1.11 |
| CHD4 | 2.04 | 1.52 |
| COL4A2 | -1.62 | 5.85 |
| CST7 | 2.78 | 3.68 |
| CTBP1 | 46.31 | 37.80 |
| CTNNA1 | 1.43 | 1.21 |
| CTSK | 1.51 | 2.68 |
| CTSL | 1.07 | 1.06 |
| CXCL12 | -1.55 | -1.08 |
| CXCR2 | 2.86 | 1.55 |
| CXCR4 | -1.56 | 3.56 |
| DENR | -1.11 | 1.38 |
| EPHB2 | 8.24 | 7.73 |
| ETV4 | 56.67 | 49.37 |
| EWSR1 | 6.55 | 4.62 |
| FAT1 | 3.71 | 2.17 |
| FGFR4 | -1.32 | 7.31 |
| FLT4 | 8.67 | -1.08 |
| FN1 | -1.48 | 57.19 |
| FXYD5 | 2.11 | 2.59 |
| GNRH1 | -1.22 | 2.31 |
| HGF | 3.06 | -1.08 |
| HPSE | -1.22 | 1.27 |
| HRAS | 1.07 | 1.12 |
| HTATIP2 | -1.48 | 1.18 |
| IGF1 | -1.48 | 7.11 |
| IL18 | 1.22 | 2.79 |
| IL1B | 32.14 | 5.26 |
| ITGA7 | 10.32 | 49.55 |
| ITGB3 | 2.49 | 12.42 |
| KISS1 | 2.32 | 1.42 |
| KISS1R | -1.48 | 8.20 |
| KRAS | 2.63 | 1.91 |
| MCAM | 3.03 | 8.63 |
| MDM2 | 3.94 | 5.63 |
| MET | 4.46 | 8.17 |
| METAP2 | 1.59 | 1.34 |
| MGAT5 | 5.10 | 8.14 |
| MMP10 | -1.15 | 1.68 |
| MMP11 | 6.81 | 8.42 |
| MMP13 | -2.39 | 7.17 |
| MMP2 | 2.10 | 28.77 |
| MMP3 | -1.43 | -60.42 |
| MMP7 | -1.48 | -1.35 |
| MMP9 | 2.55 | 2.34 |
| MTA1 | 7.52 | 11.82 |
| MTSS1 | 1.80 | 2.63 |
| MYC | 1.25 | 1.28 |
| MYCL | 3.55 | 4.10 |
| NF2 | 1.83 | 5.74 |
| NME1 | 1.25 | -1.47 |
| NME4 | 1.79 | -1.41 |
| NR4A3 | 8.49 | 2.28 |
| PLAUR | 1.56 | 2.37 |
| PNN | -1.09 | 1.14 |
| PTEN | 1.67 | -1.27 |
| RB1 | 1.28 | 1.53 |
| RORB | -28.07 | -2.53 |
| RPSA | -1.29 | 1.25 |
| SERPINE1 | 10.58 | 26.76 |
| SET | 1.56 | 2.58 |
| SMAD2 | 1.70 | -1.11 |
| SMAD4 | 1.83 | 2.15 |
| SRC | 67.72 | 111.02 |
| SSTR2 | -1.00 | 6.32 |
| SYK | -1.48 | -1.08 |
| TCF20 | 4.66 | 6.73 |
| TGFB1 | 4.08 | 7.87 |
| TIMP2 | -1.17 | 1.31 |
| TIMP3 | -8.97 | -3.75 |
| TIMP4 | 7.53 | 2.56 |
| TNFSF10 | 1.16 | 58.33 |
| TP53 | 15.93 | 19.55 |
| TRPM1 | -1.48 | 2.78 |
| TSHR | 2.56 | -2.93 |
| VEGFA | 3.41 | 1.33 |
| ACTB | 2.50 | 2.19 |
| B2M | 1.06 | -1.11 |
| GAPDH | 1.67 | 3.22 |
| HPRT1 | -1.18 | 1.10 |
| RPLP0 | 1.12 | 1.01 |
